# Time-series hyperpolarized xenon-129 MRI of lobar lung ventilation of COPD in comparison to V/Q-SPECT/CT and CT

**DOI:** 10.1007/s00330-018-5888-y

**Published:** 2018-12-14

**Authors:** Ozkan Doganay, Tahreema Matin, Mitchell Chen, Minsuok Kim, Anthony McIntyre, Daniel R. McGowan, Kevin M. Bradley, Thomas Povey, Fergus V. Gleeson

**Affiliations:** 1 Department of Oncology, University of Oxford, Old Road Campus Research Building, Roosevelt Drive, OX3 7DQ Oxford, UK; 2 Department of Radiology, Churchill Hospital, Oxford University Hospitals NHS Trust, Old Rd, OX3 7LE Oxford, UK; 3 Department of Engineering Science, University of Oxford, OX1 3PJ Oxford, UK; 4 Radiation Physics and Protection, Churchill Hospital, Oxford University Hospitals NHS Trust, Old Rd, OX3 7LE Oxford, UK

**Keywords:** Magnetic resonance imaging (MRI), Single-photon emission computed tomography (SPECT), Emphysema, Chronic obstructive pulmonary disease (COPD), Lung

## Abstract

**Purpose:**

To derive lobar ventilation in patients with chronic obstructive pulmonary disease (COPD) using a rapid time-series hyperpolarized xenon-129 (HPX) magnetic resonance imaging (MRI) technique and compare this to ventilation/perfusion single-photon emission computed tomography (V/Q-SPECT), correlating the results with high-resolution computed tomography (CT) and pulmonary function tests (PFTs).

**Materials and methods:**

Twelve COPD subjects (GOLD stages I–IV) participated in this study and underwent HPX-MRI, V/Q-SPECT/CT, high-resolution CT, and PFTs. HPX-MRI was performed using a novel time-series spiral k-space sampling approach. Relative percentage ventilations were calculated for individual lobe for comparison to the relative SPECT lobar ventilation and perfusion. The absolute HPX-MRI percentage ventilation in each lobe was compared to the absolute CT percentage emphysema score calculated using a signal threshold method. Pearson’s correlation and linear regression tests were performed to compare each imaging modality.

**Results:**

Strong correlations were found between the relative lobar percentage ventilation with HPX-MRI and percentage ventilation SPECT (*r* = 0.644; *p* < 0.001) and percentage perfusion SPECT (*r* = 0.767; *p* < 0.001). The absolute CT percentage emphysema and HPX percentage ventilation correlation was also statistically significant (*r* = 0.695, *p* < 0.001). The whole lung HPX percentage ventilation correlated with the PFT measurements (FEV_1_ with *r* = − 0.886, *p* < 0.001*, and FEV_1_/FVC with *r* = − 0.861, *p* < 0.001*) better than the whole lung CT percentage emphysema score (FEV_1_ with *r* = − 0.635, *p* = 0.027; and FEV_1_/FVC with *r* = − 0.652, *p* = 0.021).

**Conclusion:**

Lobar ventilation with HPX-MRI showed a strong correlation with lobar ventilation and perfusion measurements derived from SPECT/CT, and is better than the emphysema score obtained with high-resolution CT.

**Key Points:**

• *The ventilation hyperpolarized xenon-129 MRI correlates well with ventilation and perfusion with SPECT/CT with the advantage of higher temporal and spatial resolution.*

*• The hyperpolarized xenon-129 MRI correlates with the PFT measurements better than the high-resolution CT with the advantage of avoiding the use of ionizing radiation.*

**Electronic supplementary material:**

The online version of this article (10.1007/s00330-018-5888-y) contains supplementary material, which is available to authorized users.

## Introduction

Chronic obstructive pulmonary disease (COPD), characterized by airflow limitation and parenchymal destruction (emphysema), is currently the fourth leading cause of death worldwide, and its incidence is predicted to increase in the future [1, 2]. The conventional method of assessment of lung function in COPD is performed by using pulmonary function tests, providing an overall measure of lung function but providing limited information about regional or lobar function. The heterogeneous nature of COPD means that accurate and detailed information on regional structural and functional abnormalities are required to better characterize disease severity and enable improved choice of potential therapy, for example, lung volume reduction surgery or endobronchial valves [3]. These targeted regional therapies require robust and detailed functional and structural imaging both to support patient selection and to determine therapeutic response.

High-resolution computed tomography (CT) is widely used to assess lung structure in COPD [4, 5]. Lung parenchymal attenuation has been correlated with emphysema and pulmonary function test (PFT) measurements. Ventilation/perfusion single-photon emission CT (V/Q-SPECT/CT) has also been used to assess ventilation and perfusion in COPD [6–10]. In clinical practice, V/Q-SPECT has been shown to be a useful imaging technique in the detection of severe ventilation defects in patients with COPD [11]. Its relatively low spatial and temporal resolution may affect the accuracy of ventilation defects measured with V-SPECT [12, 13], and the radiation exposure may be a limiting factor in its use as a tool in the investigation and follow-up of patients with COPD [14, 15].

Over the last decade, the advent of non-ionizing hyperpolarized (HP) gas, i.e., ^3^He and ^129^Xe, magnetic resonance imaging (MRI), has shown considerable promise in the detection of regional ventilation defects in patients with COPD [16–20]. Although HP ^3^He exhibits intrinsically stronger MRI signal than HP ^129^Xe, the use of naturally abundant ^129^Xe is increasing due to its increased availability, lower cost, and its additional ability to be used to produce an imaging measure of gas transfer [21–23]. Conventional proton MRI of lungs, using oxygen, has also been reported to be a promising approach for ventilation and perfusion imaging [24]; however, it is limited by its inherent very low signal-to-noise ratio in the lungs [25].

Combining the increased sensitivity of HP gas MRI in the detection of ventilation defects with the high spatial resolution of thoracic CT has been reported to provide accurate quantification of lobar-specific lung ventilation [3, 26–29]. However, lobar gas ventilation measurements are not possible using CT as this only provides information about lung structure and not ventilation. Previously, Stavngaard et al compared HP ^3^He MRI to ^81m^Kr ventilation scintigraphy using a visual scoring method and showed that the large ventilation defects correlated between the two imaging modalities in healthy patients and a COPD cohort [30]. Stavngaard et al reported two main limitations in his comparison of HP ^3^He MRI and ^81m^Kr ventilation scintigraphy: (i) HP ^3^He MRI signal was influenced by the decay of magnetization and (ii) the comparison was performed using whole lung ventilation and HP ^3^He MRI. Although HP gas MRI has been shown to be sensitive in the detection of ventilation defects, the HP gas MRI signal may be influenced by multiple factors related to the gas hyperpolarization level and the decay of HP gas magnetization as a function of the number of RF pulses, flip angle (α), longitudinal decay time constant of hyperpolarization (T_1_), imaging hardware, and/or the MR sequence (i.e., k-space sampling) [31]. Recently, the development of rapid HPX-MRI imaging techniques has enabled the acquisition of time-series images in a single imaging session, in turn allowing measurement and/or correction of α and T_1_ variations [32, 33]. Using a rapid spiral k-space MRI approach, time-series images can be obtained in a single breath-hold interval allowing the measurement of α and T_1_ enabling the correction of the factors that may influence the HP gas MRI signal.

The objective of this study was to derive accurate lobar ventilation measurements using a rapid time-series HPX-MRI technique correcting the α and T_1_ variations, in a cohort of patients with COPD, and to compare them to lobar lung percentage emphysema scores measured using high-resolution CT, lobar lung ventilation/perfusion scores measured using technetium-99m diethylene-triamine-pentaacetate (^99m^Tc-DTPA) V/Q-SPECT, and lung function parameters from PFTs.

## Methods

### Subject enrollment

The study was approved by the National Research Ethics Service Committee, and written informed consent was obtained from all patients (REC approval number: 11/SC/0488). The cohort consisted of 12 patients with COPD (GOLD stages I to IV). Subject demographics, FEV_1_ (%_Pred_), and FEV_1_/FVC measurements from PFTs are detailed in Table 1. Male (*n* = 4) and female (*n* = 8) subjects enrolled had average ages of 65.6 ± 5.0 and 61.5 ± 10.8. The patients were scanned using three imaging modalities including high-resolution CT, time-series HPX-MRI, proton MRI, V/Q-SPECT, and anatomical low-dose CT images. All the scans and PFTs were performed on the same day. PFTs were performed according to ATS/ERS guidelines.

**Table 1:** Subject demographics and lung function test

Subject number	Sex (male/female)	Age (years)	GOLD stage	FEV_1_(% predicted)	FEV_1_/FVC (%)
1	Female	72	II	61	70
2	Male	67	III	41	49
3	Male	65	III	46	51
4	Female	58	II	57	68
5	Male	64	III	46	51
6	Male	73	IV	25	37
7	Male	62	II	63	63
8	Male	58	II	47	57
9	Male	64	II	63	75
10	Male	72	II	61	70
11	Female	68	III	30	47
12	Female	48	I	74	68
M ± SD		64 ± 7		51 ± 14	59 ± 12

*FEV*_*1*_, forced expiratory volume in 1 s; *FVC*, forced vital capacity

### High-resolution CT

Thin-slice (0.625 mm) CT images were acquired (Discovery 670; GE Healthcare) 60 s post-administration of 100 mL of intravenous iohexol contrast (equivalent to 300 mg of organic iodine per mL), administered as part of our approved research protocol, with the following CT parameters: FOV of 50 cm, current 50–400 smart mA, voltage of 120 kV, tube collimation of 1.25 mm, and beam pitch of 0.938:1. Images were acquired after inspiration of air in the supine position. The high-resolution CT images were used to delineate the three lobes in the right lung (i.e., right upper lobe (RUL), right middle lobe (RML), and right lower lobe (RLL)) and two lobes in the left lung (i.e., left upper lobe (LUL), left lower lobe (LLL)). The absolute CT-%emphysema score for each lobe was calculated using dedicated open-access software (Pulmonary Toolkit, PTK, version 3) representing the ratio of number of pixels less than the signal threshold of – 950 HU to the total number of pixels in each lobe as previously described [3]. The percentage CT emphysema was calculated using the equation below:

$$ \%\mathrm{Emphysema}=\left({n}_{\mathrm{pixel}}/{V}_{\mathrm{Lobe}}\ \right)\times 100 $$

where *n*_pixel_ is the number of pixels less than the signal threshold of – 950 HU and *V*_Lobe_ is the volume of lobe.

### V/Q-SPECT/CT

SPECT and low-dose CT images were acquired using a GE Discovery 670 SPECT/CT scanner according to local standard protocols. Ventilation SPECT images were acquired using 500 MBq technetium-99m diethylene-triamine-pentaacetate (^99m^Tc-DTPA), LEHR collimators, 64 × 64 matrix size, 20 s per projection with aerosol produced by a SmartVent^™^ delivery system (Diagnostic Imaging Ltd., Welford, UK). Perfusion SPECT/CT images were then acquired, low energy, high-resolution (LEHR) collimators, 128 × 128 matrix size, 30 s per projection. This SPECT/CT system has a SPECT spatial resolution of ~ 10 mm, with ~ 4 mm pixel size for a 128 × 128 matrix size. SPECT images were reconstructed using the accompanied low-dose CT for attenuation correction with commercially available software (Xeleris 3, GE Healthcare, Milwaukee, USA). SPECT/CT lobar lung ventilation and perfusion were calculated using semi-automated software (Hermes Lung Lobar Analysis version 2, Hermes Medical Solutions AB, Stockholm, Sweden) by an experienced radiologist (M.C.). The relative percentage ventilation/perfusion (i.e., relative SPECT-%ventilation and relative SPECT-%perfusion) represented the ratio of sum of signal in each lobe and volume of whole lung from low-dose CT images assuming that the sum of the lobar percentages will add up to 100%. Therefore, the absolute CT-%emphysema score was conceptually different to the relative SPECT-%perfusion and relative SPECT-%ventilation although they are both expected to correlate with the ventilation defects of COPD. The percentage lobar ventilation and perfusion were calculated using the equation below:

$$ \%\mathrm{Ventilation}=\left({S}_{\mathrm{lobe}}/{V}_{\mathrm{lung}}\right)\times 100 $$

where *S*_lobe_ is the sum of signal in a lobe and *V*_lung_ is the volume of the whole lung.

### Proton MRI

Anatomical proton MR images were acquired on a 1.5-T MR scanner (Signa HDx, GEHC, Milwaukee, WI) using the following scanning parameters: field of view 40 *×* 40 cm^2^, bandwidth 125 kHz, TE/TR 2.8 ms/1.2 ms, matrix 128 *×* 128, slice thickness 15 mm; 13 slices were acquired from posterior to anterior in the coronal plane and a single slice in axial and sagittal planes.

### HPX-MRI

Imaging was performed using 1-L bags of HP-enriched xenon gas (87% ^129^Xe) polarized to 10–15% using a commercially available polarizer (Model 9300, Polarean, Durham, NC). Time-series HPX images were acquired in the coronal plane using 13 slices with a slice thickness of 15 mm and field of view of 32 cm and constructed with an image matrix size of 128 × 128. Time-series HPX-MRI involved the acquisition of 8 sets of volume images in a single ~ 20-s breath-hold using 8-interleave spiral k-space sampling leading a time delay of ~ 2.5 s for each volume image (i.e., temporal resolution of ~ 2.5 s) and the pixel resolution of 2.5 mm (spatial resolution 2–3 mm) [32]. The time-series HPX-MRI allowed the calculation of the relative percentage ventilation (i.e., relative HPX-%ventilation) similar to the concept of SPECT relative perfusion and ventilation (i.e., relative SPECT-%perfusion and relative SPECT-%ventilation). Additionally, the absolute percentage ventilation (i.e., the absolute HPX-%ventilation) similar to the absolute CT-%emphysema score was calculated using a signal threshold method in each lung lobe.

The lobar lung masks obtained from the CT images were co-registered to proton MRI anatomical images and HPX-MRI for calculation of lobar ventilation using previously described open-source software (ITK-SNAP) [34]. Thereafter, the registered lung lobar masks applied to the HPX time-series images yielding the HPX signal time curves was performed using MATLAB. The HPX signal time curves were then fitted by the HP signal loss equations for calculation and correction of flip angle (α) and the longitudinal HP signal decay time (T_1_), as explained in Supplementary Material 1 [32]. The effects of flip angle variations on the signal dynamics and image intensity are also explained in Supplementary Material 2.

### The relative HPX-%ventilation

The percentage lobar lung ventilation relative to the whole lung volume was calculated for comparison to the relative SPECT-%ventilation and SPECT-%perfusion. The relative HPX-%ventilation was calculated as the ratio of the sum of signal in each lobe and volume of whole lung from the high-resolution CT images assuming that the sum of the lobar percentages will also add up to 100% similar to the relative SPECT-%ventilation.

### The absolute HPX-%ventilation

For comparison with absolute CT-%emphysema scores, the percentage ventilation defects were measured using a signal threshold method previously used for similar comparison between HP ^3^He MRI and CT-%emphysema score [27]. The HPX signal threshold was defined as 5% of the HPX signal measured across the whole lungs. The absolute HPX-%ventilation score was calculated as the ratio of the number of pixels below the signal threshold to the total number of pixels.

### Statistical analysis

All statistical tests were performed using GraphPad Prism (version 7.00, GraphPad Software, CA, USA). The absolute CT-%emphysema, relative HPX-%ventilation, and relative V/Q-SPECT/CT scores were compared using Pearson’s correlation with a confidence interval of 95% to investigate any significant differences. Linear regression analysis was also used to investigate the relationship between the imaging modalities for lobar measurements across the entire subject population. FEV_1_ (%_Pred_) and FEV_1_/FVC measured using PFTs were also compared to the whole lung absolute CT-%emphysema score and the absolute HPX-%ventilation for each subject.

## Results

Twelve subjects with a mean FEV_1_ (%_Pred_) = 51 ± 14 and FEV_1_/FVC (%) = 59 ± 12 as shown in Table 1 were successfully scanned using HPX-MRI and CT. The first subject was not scanned with V/Q-SPECT/CT and the second subject was not scanned with Q-SPECT.

**Fig. 1:**
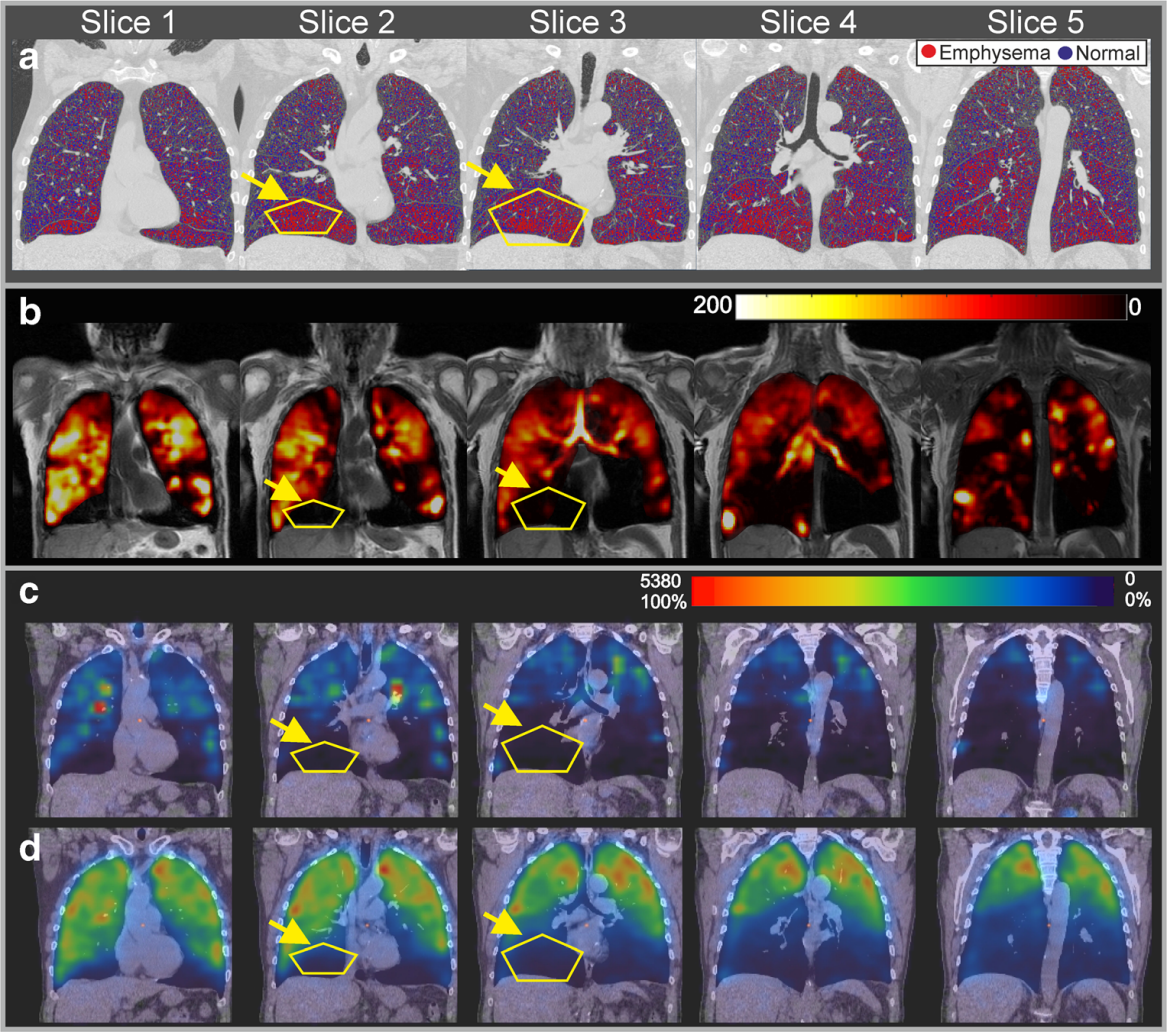
**a** The high-resolution CT images of a stage IV COPD (subject 3 in Table 1) patient for selected five slices in coronal plane including the measured emphysema values are shown. Yellow lines indicate the regions with high emphysema. **b** Proton MRI and HPX-MRI images of a stage IV COPD patient (subject 3 in Table 1) for selected five slices in coronal plane are shown. **c**, **d** V/Q-SPECT and low-dose CT fused images of a stage IV COPD patient (subject 3 in Table 1) for selected five slices in coronal plane are shown. Yellow lines indicate the regions with severe emphysema

Representative coronal slices from CT images including the emphysema map, proton MRI, HPX-MRI, and V/Q-SPECT/CT of a severe COPD subject (stages III and IV, subjects 3 and 6 in Table 1) are shown in Fig. 1a–d, respectively. The high percentage areas of emphysema (> 20%) are indicated with yellow lines on the CT in both lower lobes as shown in Fig. 1a with lung parenchyma of low attenuation (< − 950 HU) highlighted in color. Areas of CT emphysema in both lower lobes are in visual agreement with the regions of low ventilation on HPX-MRI in Fig. 1b and regions of low ventilation and perfusion on V/Q-SPECT/CT in Fig. 1c, d.

**Fig. 2:**
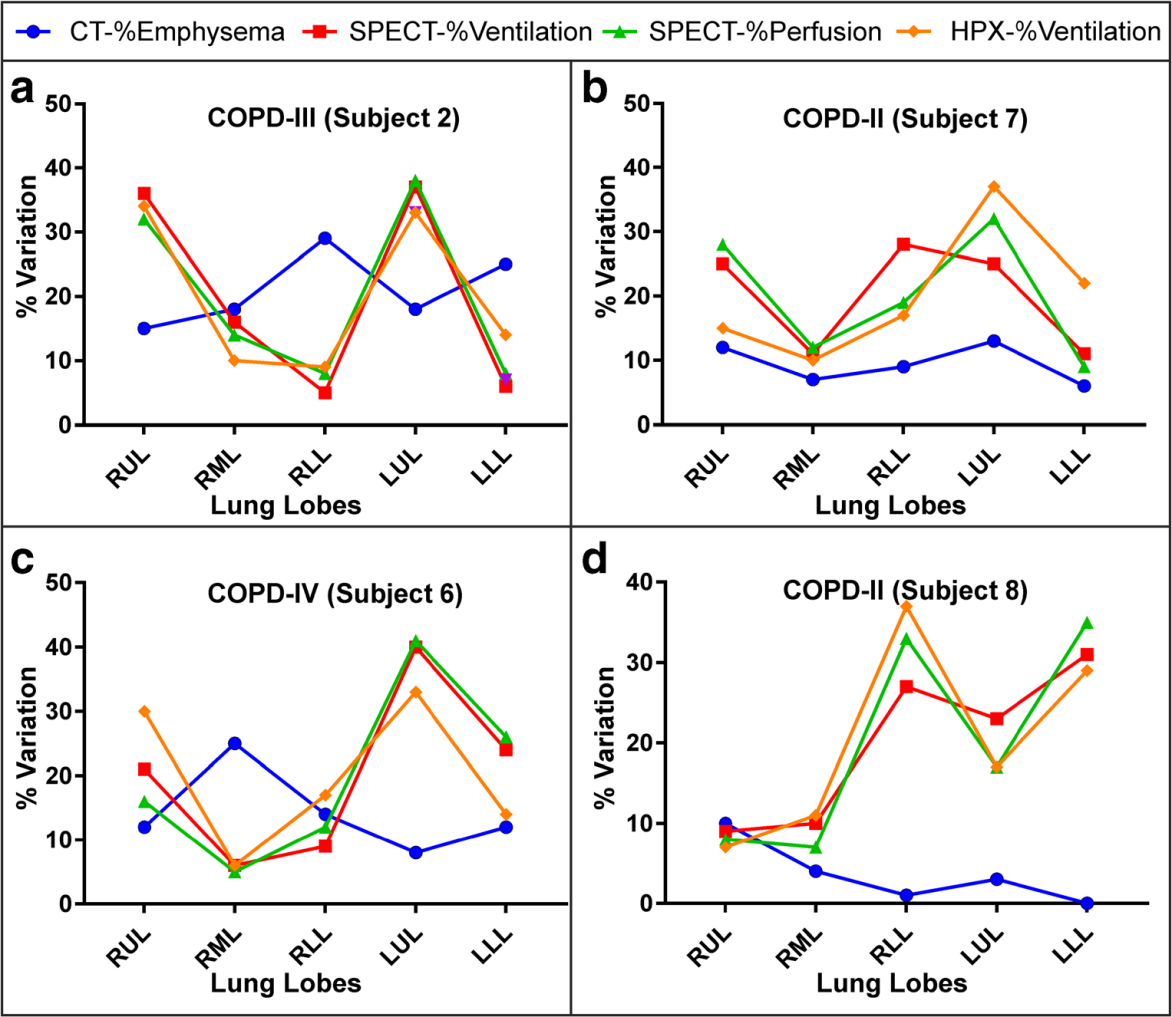
The measured absolute CT-%emphysema, the relative HPX-%ventilation and SPECT-%ventilation, and SPECT-%perfusion are shown in two severe COPD (stage III and stage IV) (**a**, **c**) and two mild COPD (stage IV) (**b**, **d**) for each lobe. RUL, right upper lobe; RML, right middle lobe; RLL, right lower lobe; LUM, left upper lobe; LLL, left lower lobe

The lobar CT, HPX-MRI, and V/Q-SPECT/CT measurements from two COPD subjects with GOLD stage IV are shown in Fig. 2a, c individually. The CT-%emphysema scores were 29% and 25% in the RLL and LLL and were greater than in the RUL = 15%, RML = 18%, and LUL = 18%. The high CT-%emphysema in the lower lobes of the lungs corresponded to conspicuous ventilation defects on HPX-MRI with the relative HPX-%ventilation of the RLL = 9% and LLL = 13%, in the V-SPECT images with the relative SPECT-%ventilation of the RLL = 5% and LLL = 6%, and in the Q-SPECT images with the relative SPECT-%perfusion of the RLL = 8% and LLL = 8%. Similarly, another COPD subject GOLD stage IV with a high CT-%emphysema was also inversely correlated to the relative HPX-%ventilation (Pearson’s correlation: *r* = − 0.856, *p* < 0.05*), SPECT-%ventilation (Pearson’s correlation: *r* = − 0.822, *p* < 0.05*), and SPECT-%perfusion (Pearson’s correlation: *r* = − 0.828 and *p* < 0.05*) in Fig. 2c. Although the inversely proportional results were statistically significant in severe COPD subjects (GOLD stage IV), there was a poor correlation between the absolute CT-%emphysema and the relative lobar measurement scores in two COPD subjects (GOLD stage II) in Fig. 2b, d, individually.

**Fig. 3:**
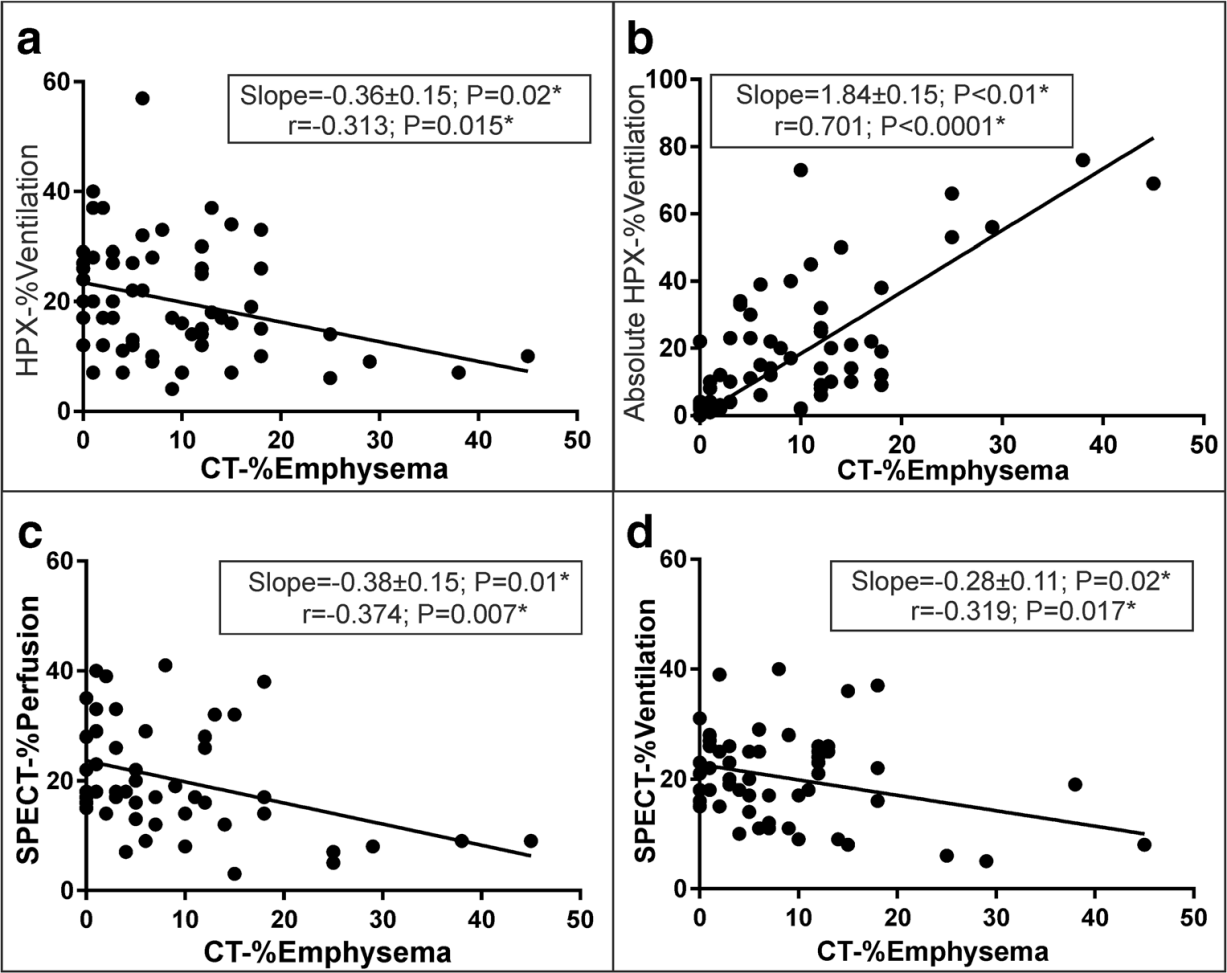
The linear regression of the lobar scores between the absolute CT-%emphysema and the relative HPX-%ventilation, the absolute HPX-%ventilation, SPECT-%perfusion, and SPECT-%ventilation are shown in **a**–**d** over the entire COPD patient population including the liner regression lines, slope, and *p* values

The linear regression of lobar measurements in all COPD subjects is shown between the absolute CT-%emphysema and the relative HPX-%ventilation, the absolute HPX-%ventilation, SPECT-%perfusion, and SPECT-%ventilation in Fig. 3a–d, respectively. The linear slopes were negative between CT-%emphysema score and the relative HPX-%ventilation (slope = − 0.36 ± 0.15, *p* = 0.02*; *r* = − 0.313, *p* = 0.015*), the absolute HPX-%ventilation (slope = 1.84 ± 0.15, *p* < 0.01*; *r* = 0.701, *p* < 0.0001*), SPECT-%perfusion (slope = − 0.38 ± 0.15, *p* = 0.01*; *r* = − 0.374, *p* = 0.007*), and SPECT-%ventilation (slope = − 0.28 ± 0.11, *p* = 0.02*; *r* = − 0.319, *p* = 0.017*).

**Fig. 4:**
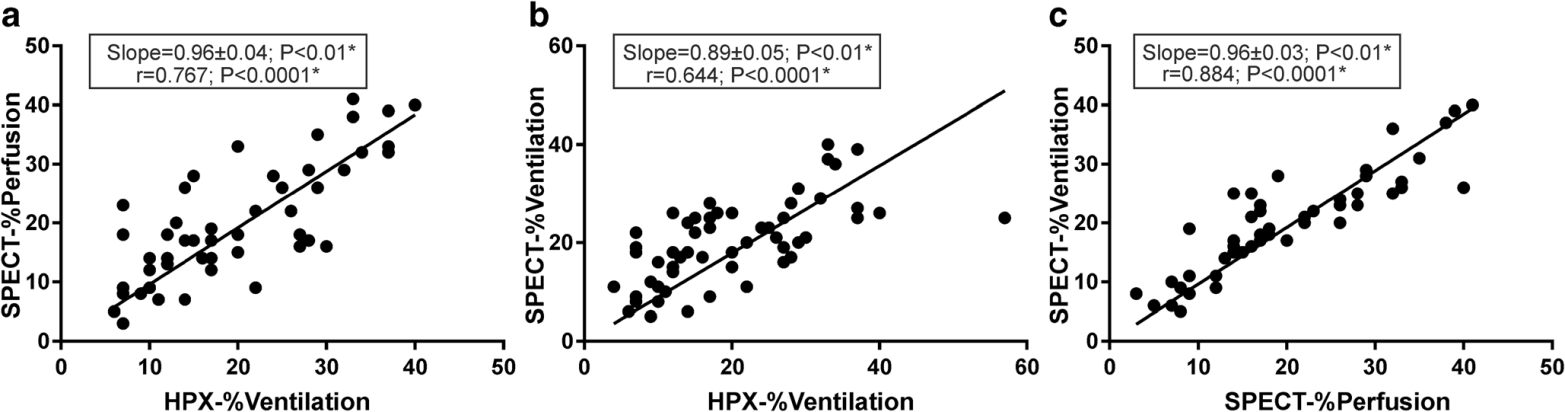
The linear regressions of the relative lobar scores between SPECT-%perfusion and HPX-%ventilation (**a**), between SPECT-%ventilation and HPX-%ventilation (**b**), and between SPECT-%ventilation and SPECT-%perfusion (**c**) are shown including the liner regression lines, slope, and *p* values

The relative measurements from HPX-MRI and V/Q-SPECT/CT are compared in Fig. 4. The correlations were *r* = 0.767, *r* = 0.644, and *r* = 0.884 with *p* < 0.001 between the relative HPX-%ventilation and SPECT-%perfusion, between the relative HPX-%ventilation and SPECT-%ventilation, and between the SPECT-%ventilation and SPECT-%perfusion in Fig. 4a–c, respectively.

**Fig. 5:**
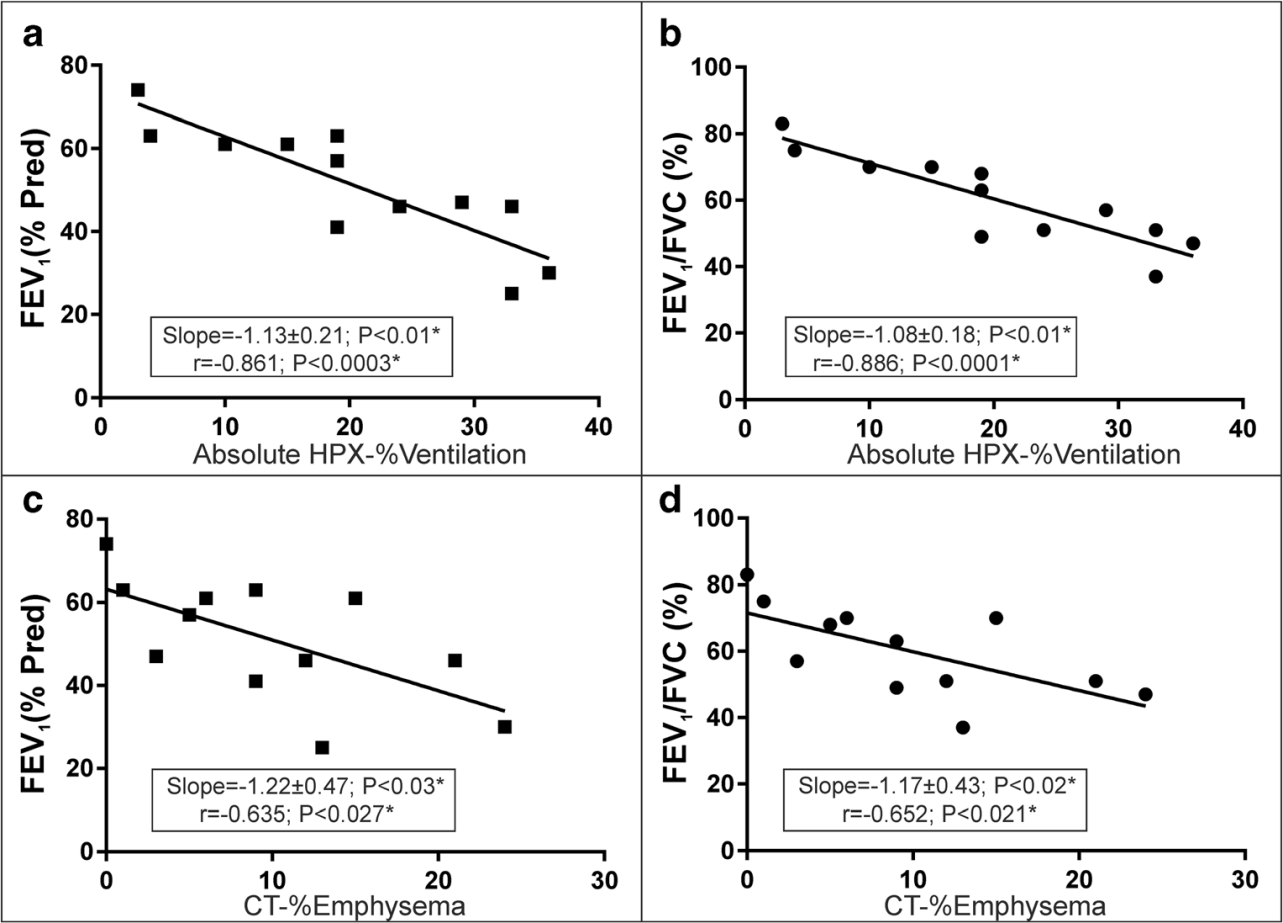
The relation of the whole lung scores between the absolute HPX-%ventilation and CT-%emphysema and FEV_1_ (%_Pred_) and FEV_1_/FVC are shown in **a**–**d** including the liner regression lines, slope, and *p* values

Figure 5 demonstrates the comparison between PFT measurements FEV_1_ (%_Pred_) and FEV_1_/FVC (%) with whole lung absolute HPX-%ventilation in Fig. 5a, b and absolute CT-%emphysema in Fig. 5c, d. There was a statistically significant correlation between HPX-%ventilation and FEV_1_ (%_Pred_) and between HPX-%ventilation and FEV_1_/FVC (%) with *r* = − 0.886 and *r* = − 0.861 with *p* < 0.001 in Fig. 5a, b suggesting a stronger correlation and then CT-%emphysema and FEV_1_ (%_Pred_) and CT-%emphysema and FEV_1_/FVC (%) with *r* = − 0.635 with *p* = 0.027 and *r* = − 0.652 with *p* = 0.021.

## Discussion

The relative and absolute lobar percentage ventilation defects obtained using a time-series HPX-MRI technique have been compared systematically to (i) the relative lobar ventilation and perfusion scores measured using V/Q-SPECT/CT, (ii) the absolute lobar emphysema score measured using high-resolution CT, and (iii) lung function (i.e., FEV_1_ (%_Pred_) and FEV_1_/FVC) in a COPD cohort. The study findings suggest that there is a very strong correlation between the lobar lung measurements of relative percentage ventilations between the time-series HPX-MRI and V/Q-SPECT/CT. Additionally, time-series HPX-MRI allowed measurement of absolute percentage ventilation of whole lung and correlates with FEV_1_ (%_Pred_) and FEV_1_/FVC measurements better than CT in a COPD cohort with GOLD stages I–IV. Compared to V/Q-SPECT/CT, the time-series HPX-MRI presents a promising non-ionizing technique for longitudinal assessment of COPD, because of its very high temporal resolution (2.5 s) and spatial resolution (2–3 mm) and as it may be employed repeatedly and only takes a few minutes to perform.

The HPX gas signal intensity directly correlates to ventilation in the lung airspaces; however, it may be influenced by other factors including the choice of k-space sampling, RF coil performance and design (i.e., surface or volume coils), and magnetic field strength [32, 35, 36]. The flip angle profiles can significantly vary over the entire imaging volume particularly for the surface RF coils as shown in Supplementary Fig. 2 resulting in variation in the signal intensities of up to a factor of 2 between the anterior and posterior regions of the RUL and LUL of the lung. Despite the HPX-MRI being acquired with a spiral k-space sampling approach using eight interleaves with relatively lower spatial resolution (pixel resolution of 2.5 mm) than the previously used Cartesian k-space sampling method (pixel resolution of ~ 2 mm) [3], the temporal resolution with the proposed technique in this study was higher by a factor of ~ 5 than the previously reported Cartesian k-space sampling method. The use of the time-series HPX-MRI technique can further improve the assessment of ventilation defects by eliminating the RF coil flip angle variations allowing relative and absolute measurements of ventilation defects. Eight time steps were used in this study for estimating the flip angle and T_1_, allowed a very high goodness-of-fit (R-squared > 0.98), as shown in the supplementary materials. In theory, using three time steps would be sufficient for solving the three unknowns as explained in the supplementary material. Although all the patients in our study were able to tolerate a 20-s breath-hold to enable eight time step scanning, the required number of images (i.e., time steps) can be reduced for the patients who are unable to manage a breath-hold interval of 20 s. Conceptually, the spatial resolution would be further increased by using less time steps and a higher number of interleaves depending on the clinical information and sensitivity required.

The negative correlation between the relative HPX-%ventilation and CT-%emphysema is consistent with previous findings reported by Matin et al [3] in a COPD cohort. As expected, the trend of correlation between HPX-%ventilation and CT-%emphysema depended on the method of HPX-%ventilation calculation (i.e., relative or absolute). There was a significant linear correlation between absolute HPX-%ventilation and CT-%emphysema, similar to a previous HP ^3^He study by Tahir et al [27, 37]. Despite the statistical correlation between CT-%emphysema score and HPX-%ventilation score, the intrinsic variations between the two imaging modalities are likely due to the sensitivity of (i) HPX to airway blockages, mucus plugs, and collapse of airways and (ii) CT’s structural assessment of the lungs as discussed previously [28, 38–40].

Although the correlation between CT and HP gas (^129^Xe and ^3^He) has been reported widely, this is the first study to our knowledge comparing lobar ventilation defects from HPX-MRI to V/Q-SPECT/CT. Stavngaard et al investigated large ventilation defects in COPD subjects using a visual (i.e., subjective) scoring factor and showed a good correlation between HP ^3^He gas MRI and regional ^81m^Kr gas ventilation scintigraphy. However, the objective computational comparison between HP ^3^He gas MRI and ^81m^Kr ventilation scintigraphy showed a relatively low correlation (*r* = 0.45, *p* = 0.016). Although there is an expected difference in diffusion between aerosol radiotracers (i.e., ^81m^Kr) and gas radiotracers (^99m^Tc-DTPA) or HP ^129^Xe gas, the measured relative lobar ventilation measures correlated better in our study between HPX-MRI and ^99m^Tc-DTPA V/Q-SPECT/CT.

As expected, HPX-MRI provided significantly higher spatial resolution and temporal resolution than V/Q-SPECT/CT. Additionally, there is image blurring in V/Q-SPECT compared to HPX-MRI images, due to motion artifact, because SPECT is performed as the patient breathes normally and the images are acquired over 30 to 40 min. In this respect, the ventilation defects identified on SPECT scans correspond to stationary ventilation defects that have stabilized after many inhalation cycles, making it insensitive to the time course of ventilation dynamics. However, HPX-MRI can capture the dynamics of xenon gas ventilation during the wash-in, breath-hold, and washout cycles separately enabling correlation with different physiological information (i.e., delayed ventilation) as discussed previously [32]. Nonetheless, the lobar lung analysis of the V/Q-SPECT images provided sufficient resolution, to confirm that the lobar ventilation and perfusion were comparable to HPX-MRI, although pixel by pixel analysis of ventilation defects may reveal greater differences between the two imaging modalities. At present a significant limitation when considering the value of HPX-MRI to SPECT/CT is that HPX-MRI is not yet in widespread use as a routine clinical imaging technique, although the body of evidence to support its use is increasing.

Although our CT scans were performed using intravenous contrast as part of our approved research protocol, calculating emphysema scores in patient with COPD does not require contrast [41]. As well as enabling emphysema scores to be calculated, it is also possible to calculate bronchial wall thickness and air flow restriction using novel CT techniques [42–44].

Although the uneven distribution of Technegas between V-SPECT and Q-SPECT is to be expected as previously reported by Bajc et al [11], the agreement between the V-SPECT and Q-SPECT measurements was greater than the agreement between HPX-MRI and V/Q-SPECT/CT. This may be because the lobar ventilation and perfusion scores in SPECT were performed using the low-dose CT data acquired in the same imaging session as the V-SPECT and Q-SPECT, potentially enabling better coregistration, whereas the lobar ventilation analysis of HPX-MRI requires image co-registration to CT performed at a different time to the HPX-MRI and is likely to be less accurate. Even though the lungs are deformable, ITK-SNAP was able to register HPX-MRI images to CT using manual segmentation, and in the future, the increased spatial resolution of conventional proton MRI may enable improved lobar segmentation, which will significantly improve the lobar analysis of HPX-MRI ventilation defects. Although in comparison, the dedicated automated software used for V-SPECT and Q-SPECT co-registration and analysis may also provide more accurate image data analysis. It is also important to highlight that the regional (i.e., pixel by pixel) comparisons from the anterior to the posterior parts of the lungs may show a larger variation with HPX-MRI than with V/Q-SPECT/CT due to differences in both the imaging processing, and the temporal and spatial resolution, and need further investigation. As such, this study raises additional questions about which imaging modality would be more sensitive to early-stage COPD when using pixel by pixel comparison of mild ventilation defects in patients with GOLD stages I–II.

## Conclusion

We have successfully demonstrated the measurement of absolute and relative percentage lobar ventilation using a novel time-series HPX-MRI sequence that corrects for intrinsic MR parameters in a small cohort of patients with COPD. This time-series HPX-MRI approach has been validated through comparison with V/Q SPECT/CT and high-resolution CT. Relative lobar ventilation measured by time-series HPX-MRI correlated statistically with relative percentage lobar perfusion and ventilation scores obtained with V/Q-SPECT/CT. Furthermore, a strong agreement between the absolute lobar ventilation from time-series HPX-MRI and the absolute lobar CT percentage emphysema score has been shown. Finally, the correlation between whole lung HPX-MRI and PFTs including FEV_1_ (%_Pred_) and FEV_1_/FVC was stronger than the whole lung CT emphysema score.

## Electronic supplementary material

ESM 1 (PDF 515 kb)

ESM 2 (PDF 173 kb)

## Abbreviations

^129^Xe: Xenon-129
^3^He: Helium-3
^99m^Tc-DTPA: Technetium-99m diethylene-triamine-pentaacetate
COPD: Chronic obstructive pulmonary disease
CT: Computed tomography
FEV_1_: Forced expiratory volume in 1 s
FVC: Forced vital capacity
HP: Hyperpolarized
HPX: Hyperpolarized ^129^Xe
LLL: Left lower lobe
LUL: Left upper lobe
MRI: Magnetic resonance imaging
PFT: Pulmonary function test
Q-SPECT: Perfusion SPECT
RLL: Right lower lobe
RML: Right middle lobe
RUL: Right upper lobe
SPECT: Single-photon emission CT
T_1_: The longitudinal decay time constant
V-SPECT: Ventilation SPECT
α: Flip angle
