# Supplementary material for: Time-series hyperpolarized xenon-129 MRI of lobar lung ventilation of COPD in comparison to V/Q-SPECT/CT and CT
Source: Eur Radiol. 2018 Dec 14;29(8):4058–67. doi: 10.1007/s00330-018-5888-y (PMC6610266; doi:10.1007/s00330-018-5888-y)
Supplement: Supplementary file 1 — (PDF 515 kb) [file 330_2018_5888_MOESM1_ESM.pdf]

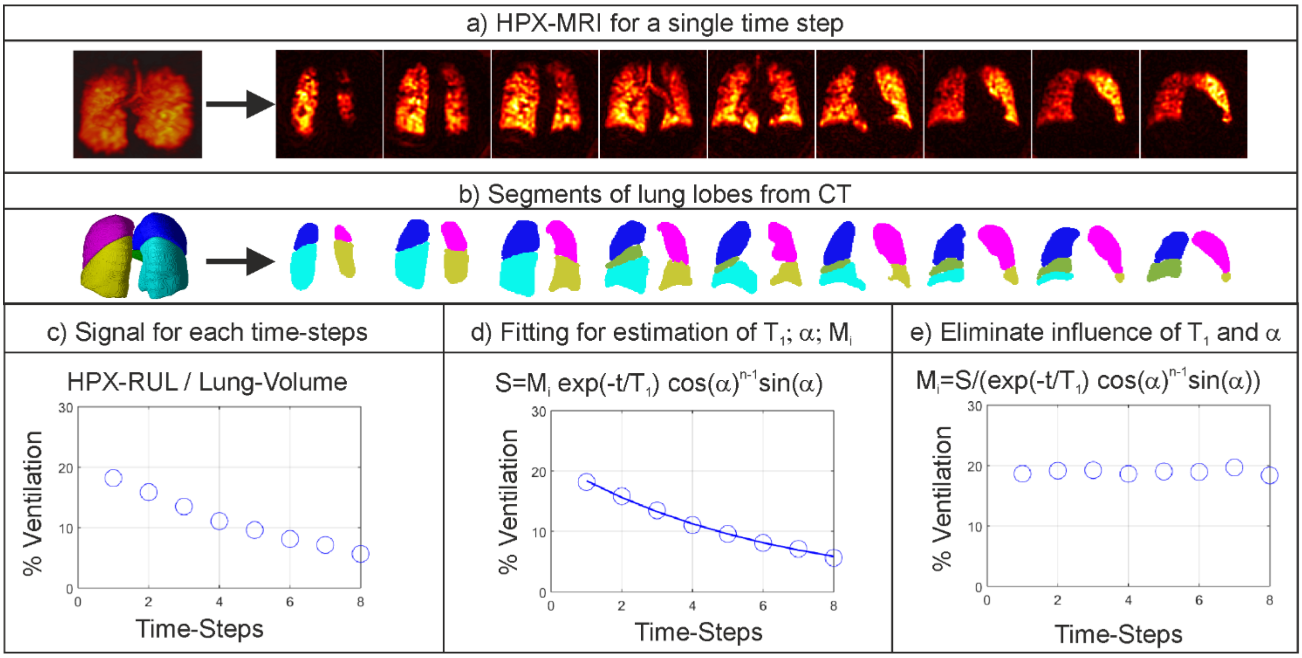

Supplementary Figure 1: Diagram of data analysis for the time-series HPX-MRI. The segments of lung lobes from the high resolution CT in (a) are applied to HPX-MRI images in (b). Signal for each time-step in (c) are fitted to the HPX signal decay equations in (d). Knowing T_1: Longitudinal magnetization decay time constant and; α: Flip angle, the signal is corrected in (e).
