# Supplementary material for: Time-series hyperpolarized xenon-129 MRI of lobar lung ventilation of COPD in comparison to V/Q-SPECT/CT and CT
Source: Eur Radiol. 2018 Dec 14;29(8):4058–67. doi: 10.1007/s00330-018-5888-y (PMC6610266; doi:10.1007/s00330-018-5888-y)
Supplement: Supplementary file 2 — (PDF 173 kb) [file 330_2018_5888_MOESM2_ESM.pdf]

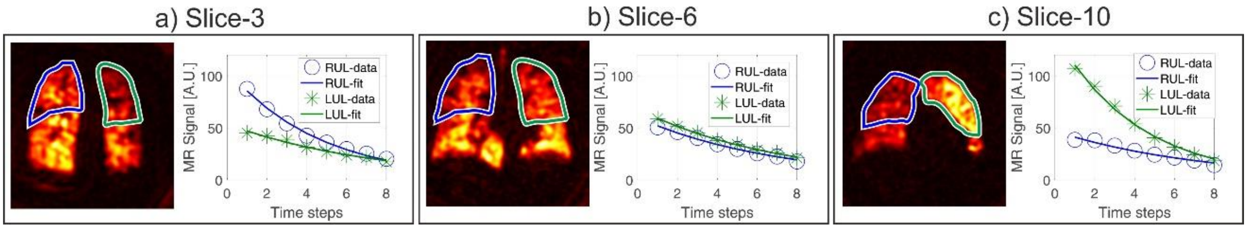

Supplementary Figure 2: HPX-MRI and corresponding signal time curves at Right Upper Lobe (RUL) and Left Upper Lobe (LUL) are shown for selected three coronal slices from anterior to posterior region of the lungs from a mild COPD subject (Subject 12 in Table 1) in (a-c). Blue and green counter lines indicate RUL and LUL respectively. The data points in the HPX-MRI signal time curves are the ratio of the sum of signal to the number of pixels.

To investigate the effects of α and T_1 to signal intensity variations, three coronal slices from an early COPD Subject (OXF0171 in Table 1) and the corresponding HPX-MRI signal time curves for Right Upper Lobe (RUL) and Left Upper Lobe (LUL) are shown in **Supplementary Figure 2 (a-c)**. The α and T_1 in **(a)** from the posterior regions of the lungs were estimated to be 9.7^o and 5.2^o; 25.9 s and 25.7 s for the RUL and LUL respectively as also shown in **Supplementary Table** 1. Although the T_1 was comparable between the RUL and LUL in **(a)**, there was an approximately two-fold difference in the signal time curves between the signal intensities at time step=1. This difference corresponded to the flip angle variations between the ROIs since the signal proportional to the flip angle (i.e. Sin(9.7^o)/Sin(5.2^o)=1.9). For the next slide in **(b)**, the α and T_1 were comparable therefore the signal time curves and the signal intensities were approximately equal between the two ROIs. In contrast, at the last slice in **(c)**, the signal in the LUL was approximately two fold larger than the RUL, due to the flip angle variation of a factor of two, as shown in the below **Supplementary Table 1**.

Supplementary Table 1: α and T_1 measurements of selected two ROIs as shown in Figure 1.

	**Slice 3**<br>α / T_1	**Slice 6**<br>α / T_1	**Slice 10**<br>α / T_1
**RUL**	9.7^o / 25.9s	6.3^o / 27.9s	5.1^o / 26.0s
**LUL**	5.2^o / 25.7s	6.4^o / 28.1s	11.5^o / 32.3s

RUL: Right Upper Lung; LUL: Left Upper Lung; α: Flip angle, T_1: Longitudinal magnetization decay time constant.
